# A machine learning-derived risk score to predict left ventricular diastolic dysfunction from clinical cardiovascular magnetic resonance imaging

**DOI:** 10.3389/fcvm.2024.1382418

**Published:** 2024-06-06

**Authors:** Qingtao Zhou, Lin Wang, Jason Craft, Jonathan Weber, Michael Passick, Nora Ngai, Omar K. Khalique, James W. Goldfarb, Eddy Barasch, J. Jane Cao

**Affiliations:** ^1^ DeMatteis Cardiovascular Institute, St. Francis Hospital & Heart Center, Roslyn, NY, United States; ^2^ Division of Cardiac Imaging, St. Francis Hospital & Heart Center, Roslyn, NY, United States

**Keywords:** machine learning, LVDD, heart failure, echocardiography, cardiac magnetic resonance imaging

## Abstract

**Introduction:**

The evaluation of left ventricular diastolic dysfunction (LVDD) by clinical cardiac magnetic resonance (CMR) remains a challenge. We aimed to train and evaluate a machine-learning (ML) algorithm for the assessment of LVDD by clinical CMR variables and to investigate its prognostic value for predicting hospitalized heart failure and all-cause mortality.

**Methods:**

LVDD was characterized by echocardiography following the ASE guidelines. Eight demographic and nineteen common clinical CMR variables including delayed enhancement were used to train Random Forest models with a Bayesian optimizer. The model was evaluated using bootstrap and five-fold cross-validation. Area under the ROC curve (AUC) was utilized to evaluate the model performance. An ML risk score was used to stratify the risk of heart failure hospitalization and all-cause mortality.

**Results:**

A total of 606 consecutive patients underwent CMR and echocardiography within 7 days for cardiovascular disease evaluation. LVDD was present in 303 subjects by echocardiography. The performance of the ML algorithm was good using the CMR variables alone with an AUC of 0.868 (95% CI: 0.811–0.917), which was improved by combining with demographic data yielding an AUC 0.895 (95% CI: 0.845–0.939). The algorithm performed well in an independent validation cohort with AUC 0.810 (0.731–0.874). Subjects with higher ML scores (>0.4121) were associated with increased adjusted hazard ratio for a composite outcome than subjects with lower ML scores (1.72, 95% confidence interval 1.09–2.71).

**Discussion:**

An ML algorithm using variables derived from clinical CMR is effective in identifying patients with LVDD and providing prognostication for adverse clinical outcomes.

## 1 Introduction

Left ventricular diastolic dysfunction (LVDD) is an important risk factor for heart failure (HF) and all-cause mortality (1, 2). Effectively and promptly identifying patients with LVDD may decrease their associated risk of adverse outcomes, improve survival, and reduce the heavy social and economic burden of heart failure (3). Echocardiography is the most commonly used modality to assess LVDD where standardized guidelines have been established and adopted for clinical use (4).

While cardiac magnetic resonance (CMR) is highly valuable in the evaluation of LV function and myocardial tissue properties, integrated assessment of LVDD using available parameters captured in routine clinical CMR examination remains challenging (5, 6), albeit individual variables derived from clinical CMR such as left atrial size and strain, LV mass and longitudinal strain are closely associated with LVDD (7, 8). In addition, measurements beyond routine CMR images have shown early promise in LVDD evaluation (9–11). The goal of the present study was to test a hypothesis that the machine learning (ML) risk score can determine the probability of LVDD using CMR variables captured in routine clinical examinations. In addition, we aimed to investigate the prognostic value of our ML score to estimate the risk of heart failure (HF) hospitalization and all-cause mortality.

## 2 Materials and methods

### 2.1 Study subjects

We identified consecutive patients who underwent echocardiography and CMR as clinically indicated with gadolinium contrast within 7 days of each other from a single center between January 2004 and December 2014. Demographic information and cardiovascular history were collected prospectively at the time of CMR. A total of 702 patients were identified initially. After excluding 96 subjects who had missing echocardiographic data for adequate evaluation of LVDD or inadequate CMR image quality using real time imaging due to arrhythmia, there were 606 subjects included in the analysis. Of those, 395 were prospectively enrolled with echocardiography and CMR obtained on the same day and the remaining 211 were identified from the clinical patient population. The study was approved by the St. Francis Hospital Institutional Review Board and a waiver was granted for the analysis of retrospective data. An additional set of subjects following the same inclusion/exclusion criterea were identified from a separate hospital within the same health system (30 miles apart representing a different catchment area) and were used as an external validation cohort. The Good Samaritan University Hospital Institutional Review Board granted approval for our use of subjects for the validation cohort and provided a waiver of informed consent for retrospective data collection.

LVDD by echocardiography was characterized following ASE guidelines (4). Details about these subjects were published in a prior study from our group (12). The outcome of HF hospitalization was identified from electronic medical records of the Catholic Health System of Long Island, NY. All-cause mortality data was obtained from both the electronic medical records and National Death Index between 2004 and 2018. All-cause mortality and HF hospitalization were combined to form a composite time-to-event outcome.

### 2.2 Imaging acquisition—transthoracic echocardiography

Comprehensive echocardiographic examination was performed using a multi-frequency transducer ultrasound system (Philips IE 33, Andover, MA) as described previously (12). Briefly, from the apical window, pulsed wave Doppler was used to interrogate mitral inflow from 3 to 5 cardiac cycles at the level of the mitral valve annulus and at the mitral leaflets' tips. Subjects with atrial fibrillation were excluded. Tissue Doppler was applied to record mitral annular velocities at the septal and lateral corners of the annulus, and the results are given as an average of both from 3 to 5 cardiac cycles. Tricuspid regurgitation velocity was recorded by continuous wave Doppler from multiple windows. Two-dimensional measurements were performed according to recommendations of the American Society of Echocardiography (13) and indexed to body surface area. Echocardiograms were utilized only for the diagnosis of presence and severity of LVDD in our analysis. Mitral inflow early (*E*) and late (*A*) peak velocities, early diastolic annular myocardial longitudinal velocity (*e*′), tricuspid regurgitation and left atrial volume (LA) index were measured in order to determine the DD grade. Subjects with indeterminate DD diagnosis (*N* = 7) were excluded; however, subjects with DD but indeterminate of grade following current guidelines were included and were considered to have DD. These subjects with DD present but indeterminate of grade were not further classified. The scan and analysis protocols were kept the same for the validation cohort.

### 2.3 Imaging acquisition—CMR

All subjects underwent CMR on a 1.5T scanner (Avanto, Siemens, Malvern, PA) with an 8-element phased array surface coil. Cine imaging of the long axis planes (2-, 3-, and 4-chamber views) and a stack of 8–12 short axis planes (contiguous 8 mm slice), starting from the mitral annulus, were acquired using balanced steady state free precession sequence with 30 phases per cardiac cycle. The average temporal resolution was 50 ms, with a typical field of view of 240 mm, flip angle of 70 degrees, repetition time (TR) of 3.1 ms and echo time (TE) 1.3 ms. Phase sensitive inversion recovery late gadolinium enhancement (LGE) imaging was performed 10–15 min after the administration of 0.15 mmol/kg of gadopentetate dimenglumine on a stack of LV diastolic short axis slices with the following parameters; TE 3.17 ms, TR = 1 × RR interval, flip angle 25°, voxel size 1.9 mm^2^ × 1.4 mm^2^ × 8 mm^3^ and FOV 360 mm^2^ × 290 mm^2^. The inversion time was selected from TI scout imaging. CMR performed on the validation cohort followed the same imaging protocol on a 1.5T scanner (Aera, Siemens, Malvern, PA) with an exception of using gadobutrol at 0.15 mmol/kg for LGE imaging.

### 2.4 Image post processing

The volumetric analysis of the cine images from CMR was analyzed using QMASS software (Version: 7.2. Medis, Leiden, The Netherlands). LA volume was analyzed following the area and length method using 2- and 4-chamber long axis cine images with commercial software (Circle Cardiovascular Imaging Inc, version: 5.11, Calgary, Canada) and indexed to body surface area (BSA). LA maximum volume (LAV_max)_ was assessed at LV end systole and LA minimum volume (LAV_min_) at LV end diastole. Regional wall motion abnormalities (RWMA) were assessed and expressed as present vs. absent. Global LV peak strain was measured in the longitudinal, circumferential and radial directions by feature tracking software (Circle Cardiovascular Imaging Inc, Calgary, Canada). The epi- and endocardial contours were drawn manually on the end diastolic phase of the 2-, 3-, and 4-chamber long-axis cine images for longitudinal strain and short-axis segmented cine images for the circumferential and radial strain and propagated automatically to calculate global peak systolic strains. All image analyses were performed by experienced operators. LGE was interpreted by cardiologists who had 6–20 years of experience. LGE was assessed as binary variable as well as characterized as either absent, ischemic or non-ischemic fibrotic patterns.

### 2.5 Machine learning algorithm and evaluation

We explored three tree-based approaches including Random Forest, XGBoost and AdaBoost. Random Forest was selected due to its superior model performance, evaluated by the highest R-squared value (data not shown).

Five Random Forest models were evaluated whose variables included (1) demographic variables only; (2) clinical CMR variables only; (3) clinical CMR + demographic variables; (4) clinical CMR + LV peak systolic strain variables; (5) clinical CMR + LV peak systolic strain + demographic variables (list of all variables was included in Table 1). Area under the curve (AUC) from the Receiver Operating Characteristic analysis was used to evaluate each model's performance. The model with the best performance was chosen to compute the ML risk score. The ML risk score was defined as the model's predicted probability of of LVDD diagnosis, conditional on the risk factors in the selected model. The Random Forest model was developed using python (v. 3.10.2) and its associated libraries [Random Forest (https://scikit-learn.org/stable/modules/generated/sklearn.ensemble.RandomForestClassifier.html); SHAP (https://github.com/slundberg/shap) and Bayesian optimizer (https://pypi.org/project/bayesian-optimization)].

**Table 1:** Baseline characteristics of the training cohort between subjects with and without left ventricular diastolic dysfunction.

	All subjects (*N* = 606)	LVDD (*N* = 305)	No LVDD (*N* = 301)	*p* values
Demographic variables				
Age (years)	66 ± 16	71 ± 15	61 ± 16	<0.001
Female (%)	377 (62%)	195 (65%)	182 (60%)	<0.001
BMI (kg/m^2^)	28 ± 6	29 ± 7	28 ± 5	<0.001
Hypertension (%)	217 (36%)	149 (50%)	68 (22%)	<0.001
Diabetes mellitus (%)	72 (12%)	58 (19%)	14 (5%)	<0.001
Hyperlipidemia (%)	217 (36%)	137 (46%)	80 (26%)	<0.001
Family history of CAD (%)	117 (19%)	57 (19%)	60 (20%)	<0.001
History of CAD (%)	87 (14%)	45 (15%)	42 (14%)	<0.001
CMR variables				
LVEDV (ml/m^2^)	85 ± 26	92 ± 31	77 ± 16	<0.001
LVESV (ml/m^2^)	42 ± 28	52 ± 35	31 ± 13	<0.001
LVEF (%)	51 ± 14	44 ± 16	57 ± 7	<0.001
LV mass (g/m^2^)	62 ± 19	70 ± 22	55 ± 12	<0.001
LVSV (ml)	84 ± 42	79 ± 38	90 ± 44	0.001
RVEDV (ml/m^2^)	72 ± 18	71 ± 20	73 ± 17	0.075
RVESV (ml/m^2^)	31 ± 17	33 ± 19	29 ± 13	0.019
RVEF (%)	53 ± 10	50 ± 12	56 ± 7	<0.001
RV stroke volume (ml)	76 ± 33	70 ± 31	83 ± 34	<0.001
LAV_max_ (ml/m^2^)	42 ± 20	45 ± 22	38 ± 17	<0.001
LAV_min_ (ml/m^2^)	25 ± 19	30 ± 21	20 ± 16	<0.001
LAEF (%)	42 ± 15	36 ± 16	48 ± 12	<0.001
RWMA Score	176 (29%)	144 (48%)	32 (10%)	<0.001
LGE prevalence	281 (46%)	194 (64%)	87 (29%)	<0.001
LGE Infarct pattern	90 (15%)	75 (25%)	15 (5%)	<0.001
LGE Non-Infarct pattern	191 (32%)	119 (40%)	72 (24%)	<0.001
LV peak strain variables				
Longitudinal (%)	−12 ± 4	−10 ± 4	−14 ± 4	<0.001
Circumferential (%)	−14 ± 5	−12 ± 5	−16 ± 4	<0.001
Radial (%)	23 ± 10	18 ± 10	27 ± 9	<0.001

A bootstrap approach was utilized to calculate 95% confidence intervals (CI). Each of the clinical datasets was stratified into five folds where four folds were used as training (80%) and the remaining (20%) as a testing dataset. This process was repeated four times. A nested cross-validation procedure (14, 15) was incorporated into the ML algorithms where the training dataset was further divided into five sub-folds and four of which were used for sub-training and the remaining for validation. In addition, a Bayesian optimizer was utilized to identify the best hyper-parameters for the random forest models. The model output for the probability of LVDD diagnosis was defined as the ML risk score.

### 2.6 Model interpretation and feature importance

We leveraged the Random Forest models with the Shapley values (16) to explore the individual contributions of the clinical variables to the probability of LVDD diagnosis. Briefly, we examine the pathway each individual subject takes through the model. The subject reaches a decision point (variable split) where the value of each contributing variable either increases or decreases the subject's probability of LVDD. Each option contributes a weight, and that weight is associated with the variables used to determine the decision point. By aggregating these decision point weights, we can identify the important features among all risk factors.

### 2.7 Statistical analysis

Continuous variables were summarized as mean ± SD and the categorical variables were expressed as numbers or percentage (%). Random forest-derived models generated scores demonstrating risk of LVDD diagnosis. Youden's index, (calculated as sensitivity + specificity − 1) was used to identify the optimal threshold for the ML risk score, which was subsequently used to stratify the subjects into having high or low ML risk scores or higher or lower probability of LVDD. Kaplan–Meier plots, generated using MedCalc (v20.110, MedCalc Software Ltd, Belgium) were developed to compare event-free survival between those with high and low probability of LVDD based on ML risk scores (evaluated with log-rank tests). Additionally, Cox proportional hazards models were created in order to evaluate the prognostic role of the probable LVDD based on our ML risk score in the context of traditional risk factors. To validate the models, we used an external dataset. The previously calibrated models based on the training dataset were tested on the validation dataset. Models were evaluated based on demographic data alone, CMR parameters alone and the combination of demographic and CMR parameters. AUC were used to validate the model performance. Analyses were performed using SigmaPlot (v14, Systat Software Inc., Palo Alto, CA). All two-sided *p* values <0.05 were considered statistically significant.

## 3 Results

Of the 606 subjects included, the average age was 66 ± 16 years and 62% were female. Of those, 305 subjects were diagnosed with LVDD by echocardiography with 113 subjects classified with grade 1, 63 grade II, 31 grade III and 98 with indeterminate LVDD grade. Compared to the subjects with no LVDD, those with LVDD were older and more likely to have cardiovascular risk factors such as hypertension, diabetes mellitus and hyperlipidemia (Table 1). In addition, the left and right ventricular ejection fractions and stroke volumes were lower, LGE prevalence was higher (64% vs. 29%, *p* < 0.001) and the LV peak systolic strain values were reduced.

**Figure 1:**
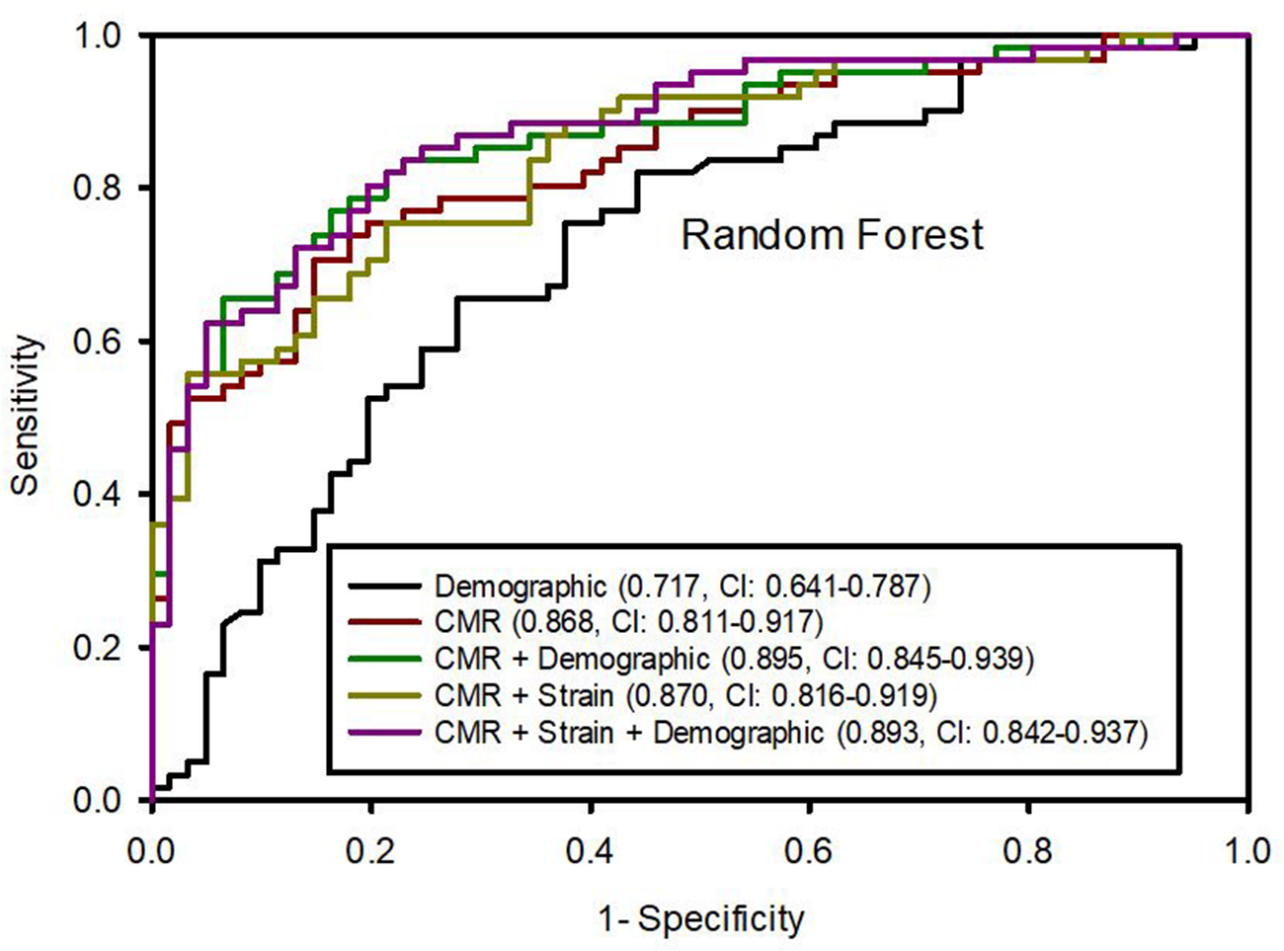
Area under the ROC curves (95% confidence intervals) from random forest algorithm predicting left ventricular diastolic dysfunction based on different variable datasets.

We tested ML model performance based on different sets of variables. The CMR variables alone were predictive of LVDD strongly with an AUC of 0.868 (95% CI: 0.811, 0.917) higher than demographic variables alone, which had an AUC 0.717 (0.641, 0.787) (*p* for comparison <0.001) (Figure 1). Combined CMR and demographical variables yielded improved model performance with an excellent AUC of 0.895 (0.846, 0.939) compared with CMR or demographical variables alone (*p* < 0.001). Conversely, the addition of LV peak strain parameters did not further improve the model performance whether in combination with CMR or with CMR plus demographic variables (0.868 vs. 0.870, *p* = 0.835 and 0.895 vs. 0.893, *p* = 0.803, respectively).

**Figure 2:**
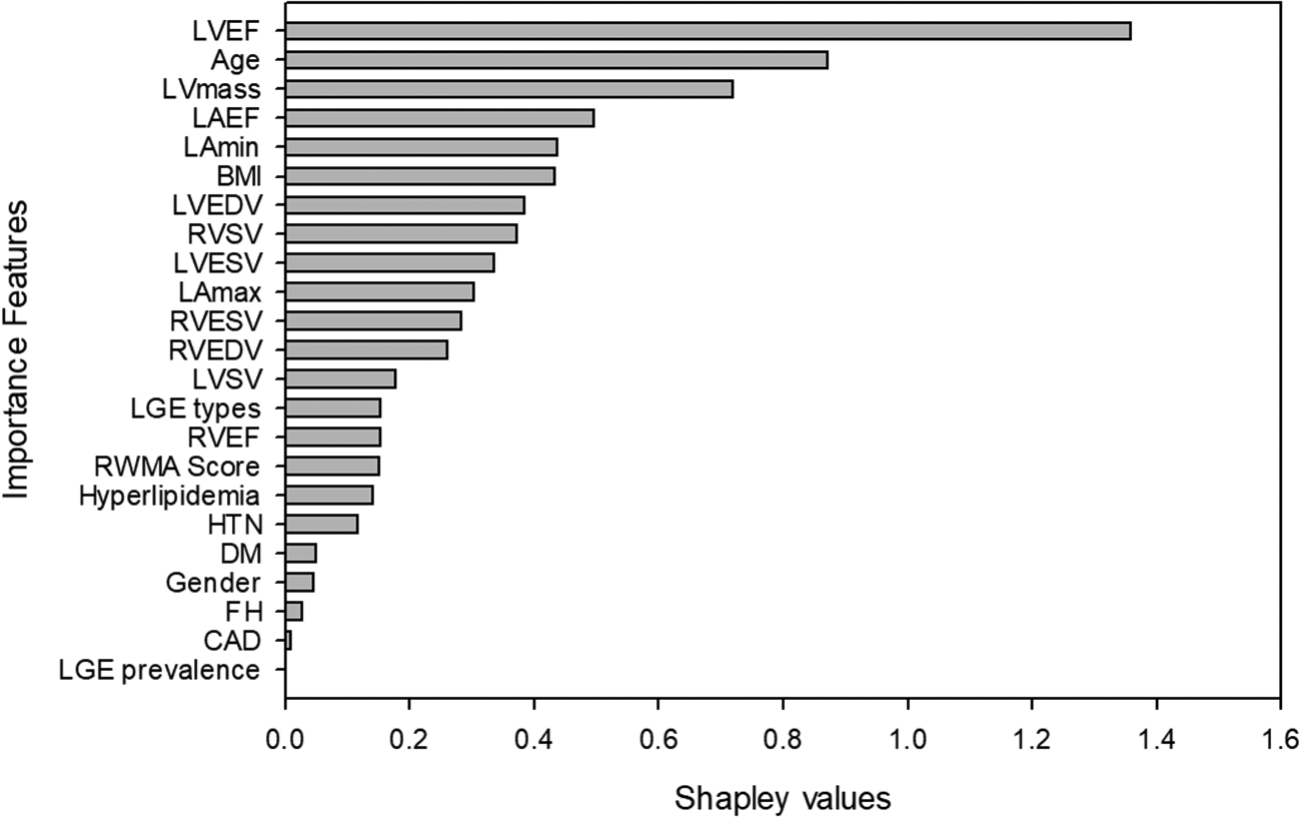
Global importance features of the random forest model.

**Figure 3:**
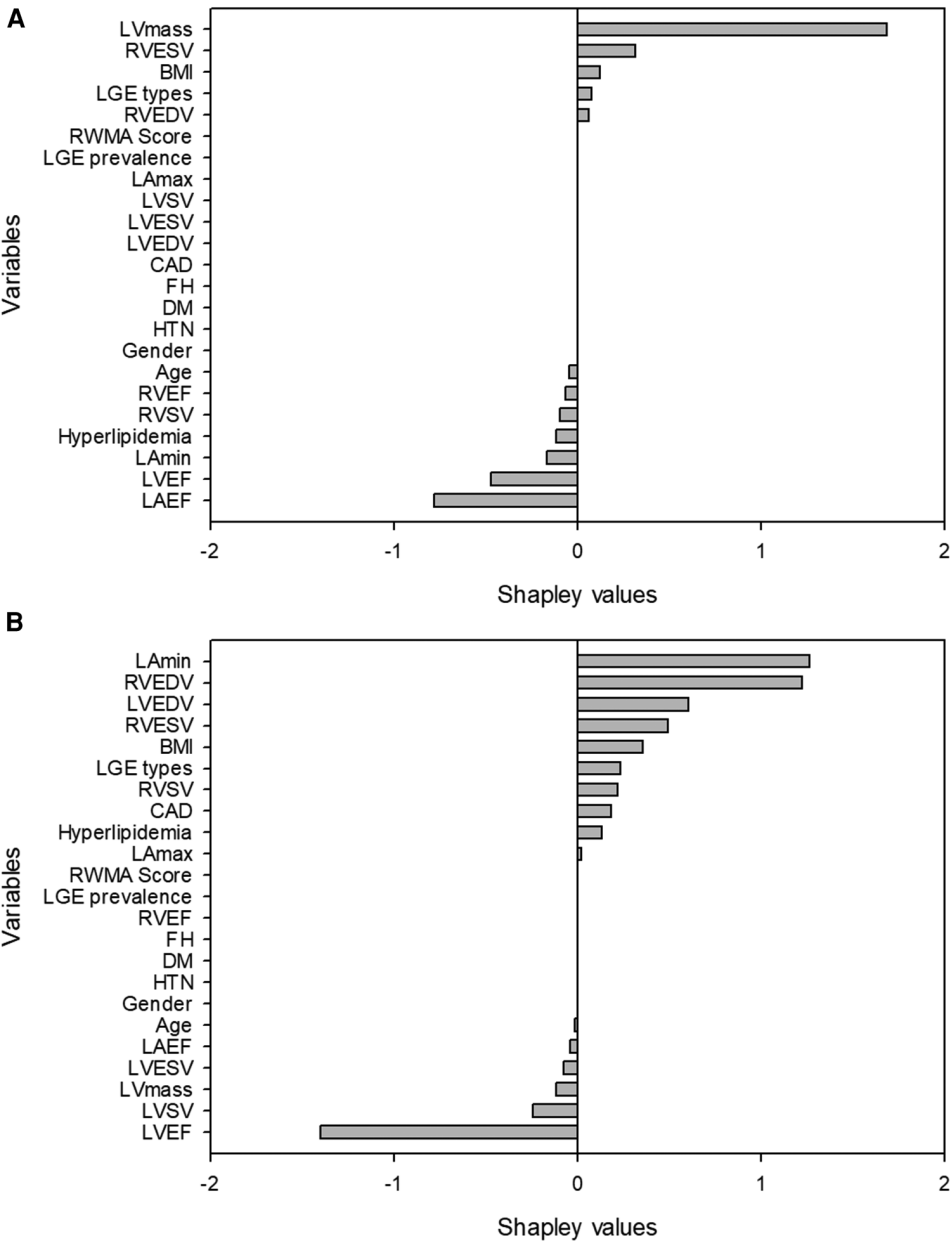
Examples of two patients with similar left ventricular ejection fraction but different machine learning scores. The first patient (**A**) was a 58 year-old female with left ventricular ejection fraction of 54%. The high machine-learning score of 0.9986 was due to unfavorable left ventricular mass, body mass index, right ventricular size and the presence of late gadolinium enhancement. The second patient (**B**) was a 53 year-old male with a left ventricular ejection fraction of 54%. The low machine-learning risk score of 0.000392 was attributed to his favorable body mass index, left ventricular mass, left and right ventricular size.

We ranked the importance features following the Shapley values based on the model with the best performance from the combination of CMR and demographic variables. The top 5 important features were LVEF, age, LV mass, LAEF and LAV_min_ (Figure 2). Next, we calculated a ML risk score for each subject. Shown in Figure 3 are two examples how Shapley values of each variable varies from subject to subject, both with normal LV ejection fraction contributing to drastically different ML risk scores.

Subjects were divided into high-risk and low-risk groups at a cut-point (0.4121) defined by Youden's index. Correspondingly, at the cut-off values, the ML risk score for identifying the LVDD were associated with a sensitivity of 0.82, a specificity of 0.80, a positive predictive value of 0.81, a negative predictive value of 0.79, and an accuracy of 0.80. Among those with low ML risk score (*N* = 309) 65 (21%) subjects were misclassified chiefly due to grade I (8%) or undetermined (11%) LVDD grade, which was 88% of all those misclassified. The remainder were due to misclassification of the advanced LVDD (grade II or III). Conversely, among those with high ML risk score (*N* = 297) 57 (19%) subjects with no LVDD were misclassified.

**Figure 4:**
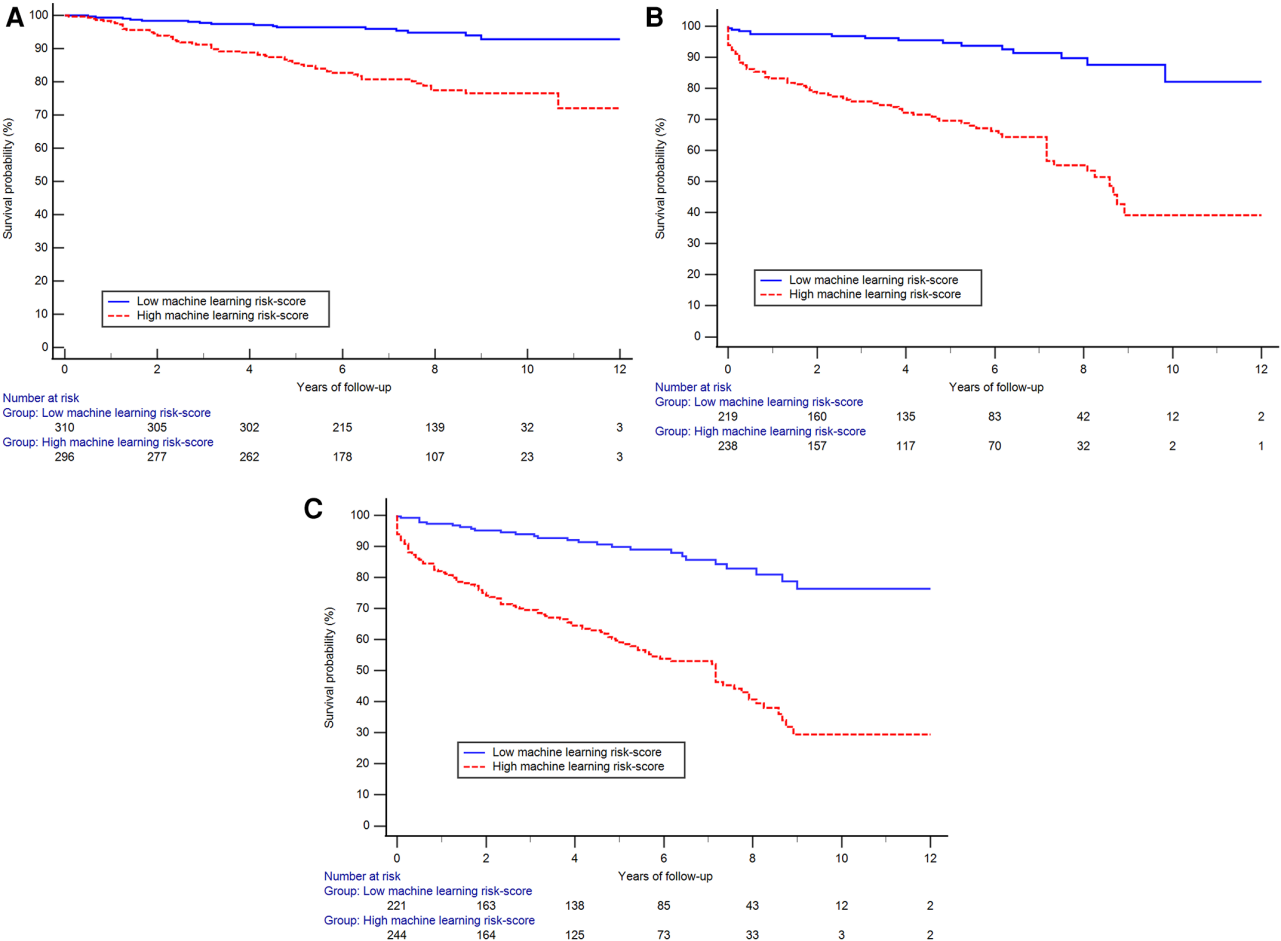
The Kaplan–Meier curves showing reduced probability of event-free survival among those with high machine-learning score than those with low score for the outcome of hospitalized heart failure (**A**), all-cause mortality (**B**) and the composite outcome (**C**) (log-rank *p* < 0.001 for all three plots).

After a mean follow up of 4.8 years, 123 subjects were hospitalized for HF, 99 died and 182 had a composite outcome of either HF or all-cause death. Subjects with higher ML risk scores were more likely to be hospitalized for HF, experience all-cause mortality, or had a composite outcome than subjects with lower ML risk scores as seen in the Kaplan–Meier curves (Figure 4) (log-rank *p* < 0.001 for all 3 comparisons). Cox proportional hazards models revealed that after adjustment for traditional risk factors including age, gender, BMI, history of hypertension, diabetes, coronary artery disease, as well as LVEF and presence of scar measured on CMR, having a high LVDD risk score was associated with a 72% increase in hazards of our composite outcome [hazard ratio 1.72, (95% CI: 1.09–2.71)], (Table 2). Additionally, compared to a model including the traditional risk factors, adding the LVDD ML risk score significantly improved model fit (likelihood ratio test *p* = 0.015).

**Table 2:** Hazards of composite all-cause mortality and inpatient heart failure admission associated with machine-learning predicted probability of LVDD.

Model^a^	Hazard ratio (95% confidence interval)
1. LVDD ML risk score per 10% increase	1.24 (1.18, 1.3)
1. LVDD high vs. low risk score	5.17 (3.48, 7.69)
2. LVDD ML risk score per 10% increase	1.18 (1.12, 1.24)
2. LVDD high vs. low risk score	3.08 (2.02, 4.7)
3. LVDD ML risk score per 10% increase	1.07 (1.01, 1.14)
3. LVDD high vs. low risk score	1.72 (1.09, 2.71)

^a^ Model 1: Risk score (unadjusted).

Model 2: Risk score + age, gender, BMI, hypertension, diabetes, history of coronary artery disease.

Model 3: Model 2 + LVEF, presence of fibrosis on CMR.

For the validation cohort (*N* = 96) the average age was 55 ± 17, younger than the training cohort of 66 ± 16 years. Participants were 41% female, fewer than the training cohort of 62%. Out of these subjects, 55 were identified as having LVDD. The subjects with LVDD were older, and had a higher prevalence of cardiovascular risk factors such as hypertension and hyperlipidemia (Table 3). Additionally, the left and right ventricular ejection fractions and stroke volumes were lower and LGE prevalence was higher. Notably the prevalence of LGE and RWMA was higher in this cohort than the training cohort, 57% vs. 46% and 79% vs. 29%, respectively. Similar to the training cohort, the model performance of the validation cohort was better using CMR variables alone than using demographic variables, AUC of 0.786 (0.710–0.856) vs. 0.724 (0.634–0.814). Combined CMR and demographic variables showed a stronger model performance with AUC 0.810 (0.731–0.874).

**Table 3:** Demographic characteristics of the validation cohort between subjects with and without left ventricular diastolic dysfunction.

	All subjects (*N* = 96)	LVDD (*N* = 55)	No LVDD (*N* = 41)	*P* values
Demographic variables				
Age (years)	55 ± 17	60 ± 15	48 ± 17	0.005
Female (%)	39 (41%)	22 (40%)	17 (41%)	0.99
BMI (kg/m^2^)	30 ± 7	31 ± 7	29 ± 6	0.235
Hypertension (%)	45 (47%)	32 (58%)	13 (32%)	0.018
Diabetes mellitus (%)	16 (17%)	11 (20%)	5 (12%)	0.46
Hyperlipidemia (%)	37 (39%)	29 (53%)	9 (22%)	0.005
Family history of CAD (%)	9 (9%)	7 (13%)	2 (5%)	0.341
History of CAD (%)	8 (8%)	7 (13%)	1 (2%)	0.152
CMR variables				
LV EDV (ml/m^2^)	101 ± 32	108 ± 35	91 ± 25	0.018
LV ESV (ml/m^2^)	53 ± 30	62 ± 36	41 ± 15	0.003
LVEF (%)	51 ± 13	48 ± 16	55 ± 6	0.008
LV mass (g/m^2^)	65 ± 21	71 ± 22	56 ± 16	0.001
LVSV (ml)	95 ± 31	95 ± 36	95 ± 24	0.943
RV EDV (ml/m^2^)	86 ± 31	89 ± 37	82 ± 23	0.289
RV ESV (ml/m^2^)	41 ± 25	44 ± 31	36 ± 12	0.143
RVEF (%)	55 ± 10	54 ± 13	57 ± 6	0.176
RV Stroke Vol (ml)	91 ± 30	91 ± 34	90 ± 24	0.839
LAV max (ml/m^2^)	47 ± 20	51 ± 20	43 ± 19	0.097
LAV min (ml/m^2^)	25 ± 23	31 ± 27	17 ± 14	0.004
LAV EF (%)	53 ± 16	48 ± 17	60 ± 12	0.0003
RWMA presence	37 (79%)	29 (50%)	8 (25%)	0.002
LGE score	4 ± 6	5 ± 6	3 ± 6	0.174
LGE presence	55 (57%)	36 (65%)	19 (46%)	0.0961

## 4 Discussion

In the present study, we trained and evaluated ML algorithms to identify patients with LVDD based on CMR variables that are routinely captured for clinical purposes. The model using only CMR variables was robust and was further improved when combined with the demographic information. The addition of myocardial strains had a neutral effect on the model performance. The model performed well on an independent external dataset. The ML risk score defining the probability of LVDD diagnosis was effective at differentiating subjects at risk of hospitalized HF and all-cause mortality.

To date, echocardiography remains the most important modality for the diagnosis of LVDD despite the complexity of the diagnostic algorithm and commonly undetermined classification. On the other hand, CMR is still limited in evaluating LVDD even though it is invaluable in assessing cardiac systolic function and myocardial tissue properties. It is worth noting that a few small CMR studies have demonstrated the feasibility of using innovative imaging or post processing techniques to evaluate LVDD (9–11, 17). Nevertheless, most of those protocols require either additional image acquisitions beyond standard clinical CMR protocols or complex post processing in need of proprietary software. In contrast, the LVDD diagnostic algorithm by echocardiography is developed based on variables that are captured during routine clinical studies. The presence or absence of LVDD can therefore be successfully defined in most cases when image quality is appropriate, albeit in a subset of patients in whom LVDD status is undetermined largely due to discordant findings among the essential variables or lack of measurement such as tricuspid regurgitation velocity when the regurgitation is absent. In the present study we tested the ML algorithm based on CMR variables that are clinically acquired, similar to the echocardiographic approach, and found the performance of the algorithm is excellent at identifying LVDD especially after combining CMR variables with common clinical variables yielding an AUC 0.895 (0.845, 0.939). More importantly, the success of the ML approach can be achieved from using variables that are clinically acquired without the need of additional image acquisitions or post processing. Interestingly, each of the top 5 most important features including LVEF, age, LV mass, LAEF and LAV_min_ have all been previously demonstrated to be associated with LVDD thereby supporting the validity of our ML algorithm (4, 7, 18). To validate the algorithm we applied the model to an independent dataset obtained from a community hospital where echocardiography and CMR were aquired by the local team for clinically referred patients, who were much more prevalent in male gender, the presence of LGE and RWMA, suggesting a different patient population from the training cohort. Nonetheless, the model performed well in predicting LVDD despite the small cohort size.

LVDD is an important condition to recognize, as it is associated with significantly elevated risk of HF and mortality. At the present time, the clinical diagnosis of LVDD is largely based on echocardiographic criteria recommended by ASE guidelines and will continue to evolve towards greater simplicity and accuracy (19, 20), which is a shared goal for the field of CMR. In this study we included only the most commonly assessed variables from clinical CMR such as cardiac chamber volumes, regional and global systolic function, and LGE, which seem to effectively support the strong performance of the ML algorithm. In addition to common clinical variables we have also examined the LV peak systolic strains in the longitudinal, circumferential and radial directions since strains have been linked to LVDD (8, 21). In our cohort, LV strains were significantly reduced in subjects with LVDD although strain impairment did not appear to further improve model performance when combined with CMR assessment. We speculate that the ML approach helps to efficiently utilize multiple clinical variables simultaneously thereby maximizing the value of each variable and consequently simplifying the evaluation. It appears feasible based on our observation, not to mention highly desirable to offer LVDD evaluation as part of the routine clinical CMR examination to compliment the assessment of systolic function and tissue characterization.

ML has often been criticized as a black box because the output data is not always easily interpretable in a clinically familiar manner. To ensure our findings are clinically relevant we applied Shapley values to change this “black box” into a “glass box” by using the explanation functions (22, 23). The Shapley values are especially helpful when they are applied to individual subjects (24). Such applications can be appreciated in two examples shown in our study where Shapley values of the same variables differed significantly between two patients, as each value is unique to the patient. Consequently, the ML risk score differentiated two patients having high or low likelihood of LVDD despite the similar LVEF between them (Figure 3). While the overall model performance of an ML algorithm is important, the true clinical value of any ML algorithm lies in the function of assessing individual patients for diagnostic or prognostic purposes. Our approach of using ML risk score is promising in assessing individual patients for the diagnosis of LVDD.

There are several limitations in the present study. The algorithm provided dichotomized probability of having or not having LVDD. The binary evaluation appears to be effective in differentiating the risk of adverse clinical outcomes providing important prognostication. However, there is lack of a grading system to further delineate the severity of LVDD, which will require additional validation and is beyond the scope of current study. We did not have invasive hemodynamis available to assist in the validation of our algorithm although LVDD may present in individuals with normal LV end diastolic pressure. While the overall performance of the ML algorithm is strong there are misclassifications in the diagnosis of LVDD. In the low ML risk score group the misclassified cases were largely made up of subjects with grade I or undetermined LVDD grade. The current guideline classifies LVDD for anyone who has reduced LV ejection fraction, an approach that continues to draw debate which may in part contribute to the misclassification. In addition, the undetermined LVDD grade by the reference standard of echocardiography is undoubtedly a source of challenge and a cause of misclassification for CMR. Nevertheless, the adverse outcome risk was significantly lower among those with low ML risk scores than those with high scores thereby supporting the validity of the LVDD diagnostic probability assessment. We included the LV peak systolic strain in the analysis. However, the diastolic strain rate would have been a better alternative in assessing LVDD although it requires additional post processing. To add variables in need of extensive post-processing would have defeated our intention to make the evaluation clinically friendly. The validation cohort size was small and the time lapse was 30 days between echocardiography and CMR as opposed to 7 days in the training cohort. Despite the constrains the ML model performed well supporting the robustness of the algorithm. Nonetheless, it is still highly desirable to have a multi-center study design with a more diverse patient population to test the generalizability of the ML algorithms.

To conclude, the proposed ML algorithm using variables derived from clinical CMR is effective in identifying individual patients with LVDD and providing important prognostic value for adverse clinical outcome assessment.

## Data Availability

The raw data supporting the conclusions of this article will be made available by the authors, without undue reservation.
